# 

**Published:** 1974-01

**Authors:** 


					Bristol Medico-Chirurgical Journal Vol 89 (i) No. 329
Contents
The Gethin Shilling
J. Jancar
A Dental Surgical Unit for the Mentally
Handicapped
J. D. Rees
Midwifery and the Egg
P. Radford
The Medical Library
Bristol Medico-Chirurgical Society ... 10
Notes and News ... ... ... 10
Printed by F. Bailey & Son Ltd., Dursley, Glos

				

